# Indies in Scotland: Exploring the Unique Role of Independent Bookshops in Scotland’s Towns and Villages

**DOI:** 10.1007/s12109-020-09759-5

**Published:** 2020-10-20

**Authors:** Audrey Laing

**Affiliations:** School of Creative and Cultural Business, Robert Gordon University, Garthdee Road, Aberdeen, AB10 7QE UK

**Keywords:** Independent bookshops, Community, Scotland, Cultural driver, Festivals

## Abstract

This project explores the business practices and cultural place of independent bookshops in Scotland. The research examines the connections that independent bookshops have with their various stakeholders, and investigates the support and policy change bookshops require in order to survive and prosper. The research finds a wealth of expertise and business acumen across Scottish independent booksellers, uniquely driven by a commitment to literacy, community and a love of books. A strong contribution to communities, welfare and social capital is identified. Policy recommendations are made, which recognise the cultural and community contributions made by bookshops, as well as proposing that bookshops be at the centre of policy planning around the regeneration of high streets.

## Literature Review

### Background and Context

This project aims to address the gap in knowledge regarding the place of independent bookshops in Scotland. For the purpose of this research, independent bookshops are understood to be independently owned bricks and mortar stores, rather than chain or online stores. While previous research explores the customer experience in bookshops [1, 11] and the role of independent bookshops in the UK [24] and Scotland [33] as well as how independent bookshops compete in Australia [16] these are disparate nuggets of academic study: there is certainly room to build more research on independent bookshops. There is little research into the role of the bookseller in practice, nor of their expertise or specialist skillset. Furthermore, there is little current literature which explores the cultural role of the bookshop.

Bookselling is currently moving through a period of digital disruption and economic challenge. Indeed, while Miller’s research from 2006 [23] suggested that many booksellers are ‘reluctant capitalists’, more recent research on UK independents from 2016 suggest that in order to survive increasingly challenging market conditions, independent booksellers now need to be ‘skilled capitalists’ [24]. The move to online purchasing, retail park shopping and the resulting threat to traditional high street shopping, is being felt across the whole of the UK [5, 14]. The increase in online purchasing is of concern to many retailers, but perhaps independent shops more than most, as they generally have less financial underpinning than chains and are therefore less able to survive difficult periods of slow sales or indeed enforced closure, as we are currently experiencing with the covid-19 crisis. Bookshops have seen a dynamic period of change in the last 25 years, from the demise of the Net Book Agreement in 1997, which allowed bookshops—and other retailers—to sell books at a price of their own choosing rather than the publishers recommended retail price, to the development of e-book readers such as the Kindle, and, most influentially, the appearance of online bookselling, in particular Amazon. Add to this the fact that books are a low profit item, and one can appreciate the challenges for independent booksellers. The aim of this research, as outlined above, is to explore how independent bookshops operate in such an environment, both from the business point of view, in terms of strategy, entrepreneurial approaches etc., but also in terms of links with local communities and how they might make some cultural or social contribution [25, 31]. This is particularly important in geographically remote settings with limited resources, therefore, most of the interviews focus on booksellers and bookshop managers in geographically isolated stores. Furthermore, the research focuses upon Scotland and Scottish independent bookshops, analysing the unique offering that Scottish bookshops make to local, often peripheral, communities.

### Independent Bookshops and the Local Community

The Scottish Centre for the Book [33] suggests that independent bookshops are ‘social and cultural hubs and provide far more to communities than books’ [33]. Indeed Pan Macmillan’s Independent Bookshop Innovation Award [26] encourages independent UK bookshops to launch innovative projects in store, with projects focusing upon ‘community outreach or supporting new readers’ particularly encouraged. This initiative assumes a symbiotic relationship between ‘community’ and independent bookshops. While there is innovative [35] and useful [33] academic work ongoing across Scottish publishing and Scottish books, there is still limited academic knowledge about the role of independent bookshops, particularly given the recent challenges of digital innovation and disruption. Previous research on bookshops has examined their roles as a ‘third place’ [25] and a restorative space [15] and any bookseller can relate tales about people spending time in bookshops for the sheer pleasure of being around books, in a comfortable and peaceful environment. O’Brien [24] argues that independent bookshops support wellbeing within their communities. Indeed this kind of ‘value creation’ in independent bookshops seems to be growing as indies are well place to encourage extra-curricular activities such as book groups or community gatherings which online book shopping cannot satisfy. The recognition of the contributions to social capital which independent bookshops can make, is acknowledged in some of the valuable nuggets of research on bookshops [11] although this presents no immediate economic reward for the bookshops. In their research, Hracs and Jansson [13] expand beyond bookshops to examine other independent shops, specifically record stores, and they place emphasis on ‘curated collections’ and expert advice ‘trusted spaces’ as advantages of these kinds of spaces. They also find that *value* is created in these settings, albeit in a non-economic sense.

Paige and Littrell’s [27] examination of craft retailers found that their definitions of success may include profit and growth, but the opportunity to educate and promote crafts to customers also featured as important values. Indeed this mirrors one of the findings from the current research—the dedication to literacy and providing a community service, of many booksellers. The curatorial, bespoke approach suggested by O’Brien [24], Hracs and Jansson [13], and Hendricks [10] highlights a commonality across these different independent retailers. Similarly, Hendricks’ [10] study of independent record stores places emphasis on the *differentness* of such a setting in comparison to the experience of an online purchase. So, the very fact that independent stores are *not* online may make them attractive to some customers. Hendricks [10] describes the meaning and ritual associated with products and services in such a setting, suggesting that customers in such a space have the opportunity to develop an affinity with an authentic community of like-minded people. Indeed there is a current resurgence in the importance of authenticity in twenty-first century life [4, 29, 30] arguably as a rejection of postmodernism and the search for real meaning in our everyday lives. The important role of place and the significance of bookshops as a destination could perhaps form the first stepping stone for the resurgence of independent bookshops, and this research proposes that the independent bookshop can play a seminal role in the redevelopment of the high street.

### Cultural Contribution

A 2017 report from the Centre for Economics and Business Research (CEBR) [7] for *The Booksellers Association* focused on the economic impact of bookshops, but also acknowledged the creative spillover impact (CSI). So, while we are used to a societal and media focus upon the economic benefits of businesses and organisations, there are other benefits to our cultural and social lives at the same time, although these often are not acknowledged or valued since they lack the immediacy of economic value in Western society. The CEBR note that the CSI benefits are difficult to define in ways that ‘facilitate measurement, quantification and monetisation’ [7]. However, they also make clear the importance of acknowledging the importance of CSI benefits ‘in order not to underestimate the importance of booksellers to the UK’ [7]. The CEBR highlights a host of benefits of bookshops for society, noting that ‘the importance of creative spillovers to the UK economy are perhaps even more significant than the current monetary economic impacts’. Some benefits of bricks and mortar stores (although not exclusively independent bookshops) include:

- Provision of a physical interface—often serendipitous in nature (rather than algorithmic)
- Events
- Interaction with literacy initiatives e.g. World Book Day
- Support of UK authors
- Careers
- Cultural hubs with events, signings, readings, comedy nights, poetry events etc.
- Impact on the high street [7].

The work of the CEBR provides useful context for the current research, validating the worth and benefits of (non-economic) benefits of bookshops, and even noting that these may be *more* significant than monetised benefits.

## Methodology

While most of Scotland’s independent bookshops are situated around Edinburgh and Glasgow, this project concentrates upon those communities which are smaller in population, since a key focus of the research is to explore any supportive, community or ‘third place’ role of the independent bookshop. This is not to deny the cultural or community role which independent bookshops play in cities; rather to concentrate upon those towns and villages which do not have branches of chain bookshops easily accessible. Stationers or coffee shops which happen to sell a small selection of books are not included in the project (although this is not always an clear distinction to make—many geographically remote bookshops must include other items in order to encourage footfall and bring in extra income in order to be economically viable). Second-hand bookshops have been excised from the study, as they are subject to different market forces, although Wigtown’s role as Scotland’s national book town is discussed.

Nine semi-structured qualitative telephone interviews were undertaken between September 2018 and January 2019. Potential interviewees had been identified from a systematic review of the Booksellers Association website, a search online looking for ‘Independent Scottish Bookshops’, and variations on that term. The first interview was with an experienced Scottish publisher who then suggested other experienced and knowledgeable independent Scottish booksellers for the study. The subsequent interviewees also suggested other potential bookshops for study, following a snowball technique [3] for gathering knowledgeable participants. There was often overlap across potential interviewees as they tended to suggest each other, despite being geographically distant. Eight of the nine interviews were recorded then transcribed and analysed for emerging themes and topics relating to the aim and objectives. Thematic analysis requires the researcher be thoroughly familiar with the data, in order to effectively identify, analyse and report patterns within the data [6]. This in turn enables the identification of themes which can then be coded. This process was a developmental one, with codes and themes being reviewed and combined where appropriate [22]. The first interview was not recorded, but expansive notes were taken and a transcript developed immediately following the interview. The proceeding findings are organised into area of thematic significance.

### Findings

This section discusses the main themes emerging from the interviews, focusing first on the positive aspects of independent bookselling, including cultural contributions and value for communities. This is followed by a discussion of the challenges and barriers to successful independent bookselling in Scotland.

## Contributions and Opportunities for Independent Scottish bookshops

### Booksellers in the Community

A key overarching theme emerging from the interviews was the dedication of the bookshop managers. In many cases, being an independent bookshop owner in Scotland seemed to be a way of life, a lifestyle choice driven by a love of books, sometimes even a drive to provide something meaningful for the local community. In other words, there is a strong argument that book retailing is different to other kinds of retail. This dedication to bookselling as a trade was often mirrored by a dedication to literacy and a strong belief in the importance of providing books for a community, often specifically children. Perhaps more than in any other retail industry, the connection between seller and product is deep and meaningful, and is certainly about much more than economic benefit. One bookshop owner commented, ‘it’s like Christmas every day!’ demonstrating his love for the trade. There is a fascination around bookselling and booksellers, and we see this in the popularity of Shaun Bythell’s books based on his bookseller experiences, e.g. *Confessions of a Bookseller*, as well as his significant social media presence on Twitter (@WigtownBookshop). The publisher interviewed, noted that many bookshops are operating at subsistence level—the owners running them are doing it for the love of books and the ‘immersive lifestyle’. As one bookseller commented wryly, ‘you wouldn’t be running a bookshop if you were only interested in the bottom line’. On a more serious note, another participant commented, that for anyone in the book trade ‘money is not the key motivator and most owners will keep going until financial returns are unworkable’. Indeed another participant noted that on occasion they order books for customers knowing they will make a loss, simply because the customer is loyal and does not want to order on Amazon.

### The Bookshop as a Cultural Driver

The publisher interviewed spoke of his many years’ experience travelling around Scotland, visiting some of the very smallest and remote bookshops, as well as the chains. Being in the position of seeing the role and impact of bookshops in some of Scotland’s most remote locations, he clearly recognised the cultural contribution of many of the bookshops, serving isolated communities and often acting as drivers for community activity and small business activity. He commented that some of the bookshop owners in the most remote communities have expert knowledge of local areas and their local customer base, and this can actually drive publication with smaller publishers—the identification of a gap in the market, a request by a customer (s) for a specific book can be filled. This specialist knowledge clearly has a cultural impact, driving publication having identified a market, and supporting smaller and local publishers.

It is important to consider that in remote rural locations, bookshops may play disproportionately important roles in the cultural life of communities. In addition to selling books, a bookshop may provide a gathering place, when no other exists, or perhaps the instore coffee shop may be a safe space or even just a place to go if there are no other nearby destinations. But sometimes the importance of a bookshop grows to become something more significant, raising the profile of a particular area and changing its identity. Following a plan to regenerate a struggling Scottish location into a book town based upon the model of Hay-on-Wye, Wigtown submitted a successful bid in the mid 1990s and has since developed a firm reputation as Scotland’s book town. It now has its own established annual book festival as well as over a dozen second hand bookshops [2]. With only around 1000 inhabitants, it is impossible that Wigtown would have organically grown into a book town, but it was economically supported to take on this role. While this study does not concentrate upon a strategically developed book town, it does nevertheless highlight that bookshops in remote communities are vital, sometimes even a lifeline for local people, as well as providing a unique destination for visitors.

### Book Festivals

As the participating publisher noted, just about every Scottish town with an independent bookshop now has a book festival. He noted the impetus that an independent bookshop can give these events, catalysing, organising and helping to push the development of book festivals, bringing them to fruition. Examples include the ‘Spirit of Moray’ festival in Elgin (population around 23,000) which has strong links with the local library as well as Yeadon’s independent bookshop. Literature Alliance Scotland [17] lists 67 Scottish book festivals in Scotland in 2020 (although many are now cancelled because of coronavirus). These take place in locations as remote as Colonsay (an island in the Inner Hebrides of Scotland, population around 124) John O’Groats (a village at the northern tip of Scotland, population around 300) and Uist (the Uists are a chain of island in the Outer Hebrides of Scotland.) Many of the bookshops studied had strong affiliations with some of the new and emerging book festivals, often in very remote and rural locations. In some cases this might involve being instrumental in the organisation and development of the book festival, and would often mean stocking the relevant books; providing the gallery/coffee shop space for launch events; supporting books signings and author visits. One owner referred to this as an ‘add-on for the community’, highlighting a common attitude amongst bookshop owners that activities might be undertaken for the benefit of the community (rather than financial benefit). Previous research has found that events such as festivals can play an important role in the maintenance and regeneration of rural communities [20, 21] and the impact of festivals and the development of ‘book towns’ is also considerable [2, 19]. While there are intrinsic challenges and difficulties associated with the development of book festivals in remote places, e.g. remote location; developing enough critical mass amongst authors in order to make their visit worthwhile, it is a challenge which is often undertaken enthusiastically by independent booksellers. It is interesting to note that in Driscoll’s 2016 research [8] into Clunes, an Australian village now a designated booktown, she notes that the very peripherality—remoteness—of book town settings ‘may offset some of the exclusivity of book culture, as the attractions of the village and its non-book-related activities enable different forms of participation and potentially open up literary culture to a broader public.’ While Driscoll here refers to book towns [2], it is worth bearing this finding in mind with regard to remote and rural Scottish bookshops. In other words, perhaps their very peripherality helps to enhance their inclusivity. The emergence of so many new Scottish book festivals demonstrates a real market for such experiential consumption, often in remote locations, perhaps part of the desire of consumers to experience *authenticity* in their consumption. Indeed, as Frank notes,—referring to book towns—‘it is the very fact that book towns exist in geographically peripheral areas that makes them places of interest to tourists’ [9].

### The Local Community

The bookshops investigated were all very active in their local communities. Indeed, if there is a way for indies in Scotland to carve a niche for themselves, and to survive the pressures of online bookselling and discounting, it may well be via this route. There was wide-ranging evidence of literary festivals; school book festivals, donation of tatty books or proof copies to schools; acting as a box office for local events—many booksellers were active in their local communities and local civic societies and many had good relationships with other local independent stores. One participant regularly gave over space in the bookshop for a book group, writers workshop and French club. One bookshop owner referred to the ‘increased professionalism of the independent bookshop sector’ and there is certainly ample evidence of such professionalism and proactivity from the participants in this study. Indeed as noted above the bookshop owners often took the initiative in driving the development of local book festivals, often hand in hand with local libraries. So it is fair to say that independent bookshops do not operate in isolation—they operate at the heart of the local community, often cooperatively with other businesses/organisations, taking the initiatives in organising cultural events. As the participating publisher commented, independent bookshops NEED to be proactive in order to generate sales, loyalty and community impact.

One bookshop owner had encountered some difficulties in getting schools events initiated at the bookshop, which she partly put down to the prohibitive costs associated with travel in rural areas. While the Scottish Book Trust [32] supports author visits via the Live Literature fund, bookshop owners still had to invest to bring such events to fruition. This particular bookshop owner had actually invested in a van in order to hold events in schools, sometimes with authors. She stated that part of her role was ‘to break barriers between authors, children and bookshops’. She noted this as a significant and important investment, especially in more deprived areas, as many children would not have met an author before. This is another example of the clear contribution that bookshops and bookshop owners, make to small, often rural communities.

One thing to note about smaller communities is what was referred to as the ‘cross-pollination’ that takes place in these communities: because there are fewer people around they may take on several different cultural/practical roles e.g. work in bookshop, volunteer in local museum and volunteer at the local school. So, people in these locations are uniquely placed to forge useful relationships and share information about what different organisations can do to help each other. One bookshop owner noted that if she ever got a visit from a local author she would also endeavour to encourage them to visit the local school and the local library. Another bookshops owner had made a financial contribution towards the development of a local cinema. This demonstrates an understanding that these kinds of facilities do not operate in isolation but work hand in hand to help develop a more vibrant village or town. As one participant said.

‘You are already invested [in the local community], as you are never going to make a lot of money. The nature of bookshop owners is that they are predisposed to being involved in local community’.

### Destination Stores and Serendipity

The main challenge in terms of economic survival for the bookshops consulted was either their very rural location, or, for those in villages or small towns, what was seen as the ‘dying high street’, a topic which has been examined in some depth, resulting in thoughtful and imaginative assessments of the problems as well as potential solutions [12, 28]. As independent shops in local high streets, such as bakers, butchers, grocers, close down there is an obvious knock-on problem of lack of passing trade for other non-essential stores, like bookshops. Books are still essentially luxury items, so there is a large part of their trade based upon ‘serendipity’ and simply passing the door.

The geographic isolation of many of the interviewees meant that their sales of books were often seasonal (April to October season was often mentioned) and visitors often had to make a specific effort to visit—so they had to make their bookshop a ‘destination store’. The local populations of the most remote stores examined in this study are around 1000, 3000 and in the larger villages and smaller towns this rose to around 10,000–20,000. The most remote bookshop had a local population of around 600 so was extremely dependent on seasonal visitors for economic survival. Many of the geographically remote stores also operated coffee shops in store, or sold craft or art items, at times on a fairly large scale in adjoining or integrated galleries. Whilst the bookshops examined varied considerably in size and scale, it seems that those that can be considered a destination providing extra value for the visitor in terms of café, art gallery, gifts, may be more successful, and also encourage the visitor to spend more time. One participant stated that the aim of his bookshop was indeed to become a destination, in part because of the significant financial investment he had made in his bookshops, which was particularly geographically isolated.

The concept of serendipity is closely aligned with the spending of time; a rare commodity for many people. Much of the survival of independent bookshops was based on this tenuous concept—the need to get customer into the shop, be able to spend time and then that time would make it more likely that books would be purchased. This was often supported by the presence of coffee shops or galleries, encouraging browsing and spending time in the bookshop. As one participant said the ‘bookshop and coffee shop work hand in hand.’ There was a suggestion that this may be a glimmer of hope for bookshops—a reaction against the focus on the instant gratification from online book purchasing may be the idea of spending time in bookshops and making the most of the serendipitous find. As another respondent noted, ‘the advantage of small places is people shop for the fun of it’.

### A Safe Space

It is difficult to imagine other private enterprises where people can visit and spend long amounts of time, relatively undisturbed, and in a safe space. One participant referred to her bookshop as a ‘community hub’, ‘a safe space’ where children could wait after school before being collected by parents. Another bookshop owner noted that his bookshop was a ‘meeting and gathering place for local people’, and that some people visited every day—especially the elderly. Some of the bookshop owners had customers who would visit specially every year on holiday. Other local customers would pop in every day. As the book publisher noted, in order for independent bookshops to be successful, many of them need that something special, or extra: they need to be a destination. As another bookshop manager noted, independent bookshops need to do something ‘more bespoke, more unique’ in order to maintain a clientele and become a destination store. This bookshop owner noted that the role of the coffee shop in her store was to encourage footfall for the bookshops, rather than income from the coffee shop itself. She went on to refer to her bookshop as a ‘social hub’ because her particular village had not previously had a café. Indeed, she referred to her bookshop as a tourist attraction, good for the local economy, especially at seasonally busy times. She said ‘on a social level, the café is critical in terms of social cohesion’.

## Challenges and Barriers for Independent Bookshops

### Amazon

There was frequent mention of Amazon from the interviewees, and there is no doubt online bookselling is one of the biggest changes to the bookselling industry in recent years. There was particular frustration at the tax breaks awarded to Amazon [36] for their provision of a large number of jobs in their distribution warehouse. This approach of offering tax breaks to large organisations focuses upon immediate economic and employment impact, but has unfortunately a negative knock-on effect upon bookshops who do not receive these financial breaks and opportunities. As well as encouraging the cannibalisation of the book trade (moving book buyers from bricks and mortar to online book purchase), it fails to recognise the important multi-faceted role played by local bookshops, which often serve as a cultural hub or a gathering place for local people, visitors and service a broad geographical area. Amazon was generally perceived as lacking the subtleties and benefits of local bookshops. These benefits can be difficult to quantify in terms of economy, but are more easily understood in terms of cultural value for remote locations [7].

### Relationship with Schools and Libraries

Historically, booksellers have developed good cooperative relationships with librarians in local school libraries as well as public libraries in the local community. Librarians have purchased books from the bookshops, developing a symbiotic relationship where the bookseller shares specialist knowledge with local stakeholders. However, a move to streamline the procurement process—often using national book wholesalers—has resulted in a more financially efficient system which at the same time has degraded the strong communication network across bookshops, schools and libraries. This kind of tight knit community network is an important one, especially in smaller communities, but it has often been lost in the drive towards immediate economic efficiency. As one respondent noted ‘I’m not saying it’s deliberate, but no-one’s ever thought it through…’ A long-term strategy from local councils as well as the Scottish Government, which acknowledges small independent businesses and their role in rural locations, was noted as lacking.

### Local Councils

There was mixed feedback around local councils, councillors and politicians. At times, individual councillors had been very supportive of small bookshops, but often, booksellers found bureaucracy infuriating and counter-intuitive. Asked about the effect of the local council, one interviewee relied ‘Crap. I would say they actually have a negative effect on the success [of this area].’ One respondent commented that the council had ‘no understanding of issues in rural areas.’ Nevertheless, on an individual level, some bookshop owners had quite positive relationships with council members. For example one bookshop owner is involved with the council in terms of school events and works with a liaison officer at the council to help organise a teachers’ reading group at her shop. Business rates is another contentious issue for independent bookshops, although it is worth acknowledging that this problem is not exclusive to Scottish bookshops [18]. Some of the bookshops in this study qualified for full business rate relief, but some were just above the threshold for qualification (based on rateable value) and this presented a significant financial burden given the limited turnover of these small businesses. One bookshop owner commented that their annual business rates had been increased by almost 100% year on year. They duly appealed and waited a year to hear that the appeal had been unsuccessful. With only three part time members of staff, the bookshop did not have the resources to challenge the judgement. There was a lot of frustration with the business rates system, including the time to progress appeals against rating decisions.

### Scottish Government

There is a drive in the Scottish Government to improve literacy and numeracy, evidenced by various initiatives: *Every Child a Library Member* [34] was launched in 2015 by Scotland’s First Minister Nicola Sturgeon and was designed to introduce automatic library membership to Scottish children. *The First Minister’s Reading Challenge* was a further initiative, running in 2017 to encourage children to read as many books as possible [33]. However, while there is a clear connection made between literacy and libraries and schools in these documents, the connection with bookshops and their vital role in the support and encouragement of literacy, is absent. Indeed, gripes from the bookshop managers with the Scottish Government include the perceived lack of integration between bookshops and the SNP strategy to increase literacy and childhood reading. This was seen as an opportunity missed, especially given the clear dedication to literacy and learning evidenced by many bookshop owners. Nevertheless, many of the bookshops examined took an extremely proactive role in the encouragement of author visits. As referred to earlier, one bookshop owner had invested in a van in order to ‘share’ visiting authors with local schools. As one bookshop manager noted, when organising author visits, she would always mention other bookshops in the area, and referred to a ‘collegial’ sense among bookshops in the broad geographical area. This approach to ‘sharing’ visiting authors across bookshops, schools and libraries is perhaps easier to do in a relatively remote community since, in this instance, although they all sell books, they are about an hour’s drive apart so are not in direct competition. As one frustrated bookshop owner said of the Scottish government, ‘They don’t *help* us! At *all*!’.

### The Impact of the Net Book Agreement

Reflecting upon the current situation of challenges for Independent bookshops in Scotland a topic raised by many interviewees was the demise of the Net Book Agreement (NBA). The NBA cessated in 1997, following the growth in discounting by large chain bookshops. At that point, it was impossible to foresee the emergence of online bookselling and the dominance of Amazon which, along with the demise of the NBA, combined to form a three-pronged attack on independent booksellers, which many failed to survive. The NBA had previously ensured that books were all sold at the same fixed retail price, and books could not be discounted. The current situation of discounting, special ‘bulk buy’ deals with publishers, and ‘loss leaders’ – the practice of deliberately selling books at a very low price in order to draw in customers—were all initiatives almost exclusively available to chains. Independents rarely had the financial clout to negotiate such deals, or to withstand sudden financial pressures. The ongoing impact of the demise of the NBA, and the continued growth of online bookselling has left many independent bookshops in a parlous state. As one participant noted ‘we are on the cusp of vulnerability’—many bookshops were just one poor season or one unexpected bill away from closure. One bookseller noted that it is sometimes cheaper for her to buy new books from the local supermarket than it is to get them direct from the book wholesaler or publisher. As has been noted many times before, it is difficult to think of any other product which, when it is new, is vastly discounted. As one bookshop owner said, what they want is a level playing field, so if the chains get special discounts or signed copies from publishers, why not the independents too?

## Conclusion, Implications and Recommendations

### Community and Cultural Contribution

There is a lack of integrated strategy at council and governmental level with a focus on immediate economic benefits but a lack of foresight regarding longer term impact, especially cultural and community value. For example, the symbiotic relationship between bookshops and school/library supply; scope to recognise and reward bookshops as community hubs or safe spaces; recognition of the cultural contribution made by bookshops in organising festivals, author visits, events; providing community space for book groups/schools etc. It seems clear that neither national nor local government recognise the cultural value of bookshops in remote towns and villages. Indeed this is a particular issue for remote independent bookshops in Scotland. The economic contribution of large employers like Amazon are more immediately obvious, and they are therefore in receipt of substantial tax breaks, but the less visible work and contribution to local communities in terms of support of the high street, working in tandem with other small businesses and cultural contributions such as literacy and even providing a ‘safe space’ tend to be less easy to categorise in terms of economic value [7]. However, collectively the contribution made to communities both culturally, and societally, is significant. Many of the interviewees take an extremely proactive attitude towards their local area, community and high street and are real catalysts for cultural development and community value. Much of the work undertaken by independent bookshops is at a small scale but collectively it is extremely powerful. This research has found many independent bookshops to be hubs of activity, proactivity and philanthropy; safe spaces for communities; protectors and promoters of literacy and most of all, lovers of books.

### Future Potential

Where high streets exist (e.g. in small towns and villages) the proactive engaged activism of bookshop owners can potentially be captured to help redevelopment of the high street. As noted earlier, there are many imaginative proposals for the ‘high street problem’ [12, 28]. This includes the promotion of destination shops, experiential venues which must encapsulate fantastic experiences, places where people want to spend time. Bookshops fit this idea perfectly. It is important to realise that a realistic future for high streets must focus upon social, cultural and experiential destinations rather than solely a ‘place to purchase’ and these values are completely in line with those bookshop owners and managers interviewed here. The bookshop managers recognised that they must offer something different, something extra in order to survive. As one participant noted, ‘bookshops give identity to a high street’. Alongside cafes, artisan stores and other destination venues, independent bookshops can be the heart of the high street.

There are specific barriers in place for independent bookshops which are outwith their control. Considered policy change would remove or alleviate these barriers.

### Policy Recommendations

1. An overhaul of the business rates system in order to recognise the cultural contribution of independent bookshops would ensure that those bookshops that just miss out on business rate relief were protected from going out of business. There is no consistent nationwide application of business rates, and the regional variations can have serious consequences on small business owners. As noted above, many booksellers operate on subsistence rates and the impact of an uplift in business rates can kill off a business.
2. A percentage of council budgets should be dedicated to small bookshops (small businesses) in recognition of their contributions to local cultural activity, and also to promote them as cultural hubs, community contributors, destination stores, safe spaces and contributors to tourism. For example, a substantial increase in Live Literature funding [32] accessible to bookshops to incentivise more author events.
3. Libraries and schools should be mandated to produce a certain number of author events through the year with authors and stock sourced through local bookshops. Not only would this result in educational benefits for the local community, it would also strength the symbiotic relationship between schools, libraries and bookshops.
4. Serious pursuance of tax avoidance should be undertaken, given small independent bookshops are not usually in a position to hire accountants in order to avoid tax, unlike larger organisations which can routinely avoid this by having offshore/remote tax addresses. The fact that tax breaks exist for large businesses who contribute little to local cultural value, is a bitter pill to swallow for small bookshops who contribute so much to local communities. Business support grants should not be given to tax avoiding companies or companies who use retail price predation to drive others out of business.

Finally, this research is not intended to be comprehensive or an end in itself: Rather it should be seen as a starting point for further research and debate, perhaps building upon and expanding the content around the key concerns and issues, as well as the unique skills of independent booksellers. This article may also be used as a starting point for exploring related ideas or comments from the public or other independent booksellers, perhaps those based in towns and cities, which are not examined in this study. It is hoped that this paper will also encourage information sharing and the development of a knowledge community across Scottish independent bookshops.
